# Left ventricular function recovery in peripartum cardiomyopathy: a cardiovascular magnetic resonance study by myocardial T1 and T2 mapping

**DOI:** 10.1186/s12968-019-0590-z

**Published:** 2020-01-06

**Authors:** Yao-Dan Liang, Yuan-Wei Xu, Wei-Hao Li, Ke Wan, Jia-Yu Sun, Jia-Yi Lin, Qing Zhang, Xiao-Yue Zhou, Yu-Cheng Chen

**Affiliations:** 1 Department of Cardiology, West China Hospital, Sichuan University, No.37, Guo Xue Xiang, Chengdu, Sichuan 610041 People’s Republic of China; 2 Department of Cardiology, Beijing Hospital, National Center of Gerontology, Beijing, China; 3 Chinese Academy of Medical Sciences and Peking Union Medical College, Beijing, China; 4 Department of Geriatrics, West China Hospital, Sichuan University, Chengdu, Sichuan China; 5 Department of Radiology, West China Hospital, Sichuan University, Chengdu, Sichuan China; 6 MR Collaboration, Siemens Healthineers Ltd., Shanghai, China

**Keywords:** Peripartum cardiomyopathy, Cardiovascular magnetic resonance, Extracellular volume, T1 mapping, T2 mapping, Left ventricular function recovery

## Abstract

**Background:**

Peripartum cardiomyopathy (PPCM) is rare and potentially life-threatening; its etiology remains unclear. Imaging characteristics on cardiovascular magnetic resonance (CMR) and their prognostic significance have rarely been studied. We sought to determine CMR’s prognostic value in PPCM by using T1 and T2 mapping techniques.

**Methods:**

Data from 21 PPCM patients from our CMR registry database were analyzed. The control group comprised 20 healthy age-matched females. All subjects underwent comprehensive contrast-enhanced CMR. T1 and T2 mapping using modified Look-Locker inversion recovery and T2 prep balanced steady-state free precession sequences, respectively. Ventricular size and function, late gadolinium enhancement (LGE), myocardial T1 value, extracellular volume (ECV), and T2 value were analyzed. Transthoracic echocardiography was performed at baseline and during follow-up. The recovered left ventricular ejection fraction (LVEF) was defined as LVEF ≥50% on echocardiography follow-up after at least 6 months of the diagnosis.

**Results:**

CMR imaging showed that the PPCM patients had severely impaired LVEF and right ventricular ejection fraction (LVEF: 26.8 ± 10.6%; RVEF: 33.9 ± 14.6%). LGE was seen in eight (38.1%) cases. PPCM patients had significantly higher native T1 and ECV (1345 ± 79 vs. 1212 ± 32 ms, *P* < 0.001; 33.9 ± 5.2% vs. 27.1 ± 3.1%, *P* < 0.001; respectively) and higher myocardial T2 value (42.3 ± 3.7 vs. 36.8 ± 2.3 ms, *P* < 0.001) than did the normal controls. After a median 2.5-year follow-up (range: 8 months-5 years), six patients required readmission for heart failure, two died, and 10 showed left ventricular function recovery. The LVEF-recovered group showed significantly lower ECV (30.7 ± 2.1% vs. 36.8 ± 5.6%, *P* = 0.005) and T2 (40.6 ± 3.0 vs. 43.9 ± 3.7 ms, *P* = 0.040) than the unrecovered group. Multivariable logistic regression analysis showed ECV (OR = 0.58 for per 1% increase, *P* = 0.032) was independently associated with left ventricular recovery in PPCM.

**Conclusions:**

Compared to normal controls, PPCM patients showed significantly higher native T1, ECV, and T2. Native T1, ECV, and T2 were associated with LVEF recovery in PPCM. Furthermore, ECV could independently predict left ventricular function recovery in PPCM.

## Background

Peripartum cardiomyopathy (PPCM) is a rare, life-threatening pregnancy-associated disease that is typically marked by ventricular dysfunction. The incidence of PPCM is gradually increasing [1] and varies geographically, most likely due to socioeconomic and genetic factors [2]. Previous studies have reported the incidence rates of the disease in different geographical regions as follows: 1 per 300 live births in Haiti [3], 1 per 346 live births in China [4], 1 per 1000 live births in South Africa [5], and 1 per 900–4000 live births in the United States [6–8]. Although viral myocarditis, abnormal immune responses to pregnancy, abnormal responses to hemodynamic stress during pregnancy, cytokine-induced inflammation, and genetic factors have been suggested to be involved in the pathogenesis of PPCM, the precise pathogenetic mechanism remains unclear [9]. The reported outcomes vary from recovery of the left ventricular (LV) ejection fraction (LVEF) within 6 months of diagnosis in 73% of the study patients [10] to the need for heart transplantation or even death [11, 12]. The lack of prognostic indicators in PPCM patients is another important clinical challenge [13].

Cardiovascular magnetic resonance imaging (CMR) is a recognized radiation-free technique for assessing cardiac structure and function and myocardial tissue characterization. Several case series and reports have shown that CMR late gadolinium enhancement (LGE) has potential value in predicting poor clinical outcomes in PPCM patients [11, 14, 15]. However, two recent multicenter and nationwide studies showed that LGE at the baseline and during follow-up were uncommon (only 3.6–14%) in PPCM [16, 17]. Furthermore, patients who did not have LGE also showed poor clinical outcomes [18]. This suggests that LGE alone has limitations as a prognostic indicator in PPCM patients. Furthermore, two case series showed myocardial edema by using T2-weighted images in PPCM patients [18, 19], while another case series showed no obvious edema on T2-weighted images [14]. This may be due to the inherent limitations of T2-weighted imaging or poor image quality [20]. CMR with native T1 and T2 mapping offers a unique noninvasive way to quantify myocardial diffused fibrosis, measure extracellular volume (ECV), and detect myocardial edema. A recent CMR study with T1 mapping showed that LV remodeling is associated with myocardial hypertrophy, but not with edema or diffuse fibrosis of the myocardium or LV contractile dysfunction during normal pregnancy [21]. However, no study has analyzed myocardial tissue characterization in PPCM by native T1 and T2 mapping or its predictive value for outcomes in PPCM patients. Therefore, the objective of our study was to analyze myocardial tissue characterization on CMR with native T1 and T2 mapping techniques and determine their potential value for predicting LV function recovery.

## Methods

### Study population

During the period from August 2013 to June 2018, 21 new-onset PPCM patients (presence of typical heart failure (HF) symptoms ≤6 months) in our CMR registry nonischemic cardiomyopathy cohort were included. No patient had a history of cardiovascular disease before developing PPCM. No abnormality had been found in the electrocardiogram (ECG) or physical examination during pre-pregnancy examination. The onset times for abnormalities ranged from the last month up prior to delivery to 5 months postpartum and fit into the strict criteria of PPCM established by the European Society of Cardiology [13]. All patients underwent CMR scan after the termination of pregnancy. The interval between CMR and onset time ranged from 7 days to 3 months, with a median of 1 month. Follow-up was performed by a review of the electronic databases and telephone interviews. An LVEF of ≥50% by transthoracic echocardiography follow-up after a minimum of 6 months was defined as LV function recovery. Ten of the enrolled PPCM patients underwent repeated CMR imaging during the follow-up. We identified 20 age-matched healthy females, without a history of hypertension, diabetes, or any other known heart disease, as the control group.

### CMR data acquisition

#### Scanning protocol

CMR examination was performed with a standardized clinical protocol [22] using a 3 T CMR scanner (MAGNTEOM Trio, Siemens Healthineers, Erlangen, Germany) with an eight-channel phased-array body coil. All images were acquired with ECG-gating and breath-holding. The imaging protocol consisted of a balanced steady-state free precession (bSSFP) cine sequence for left and right ventricular function, an inversion recovery turbo-flash pulse sequence after gadolinium administration for LGE, a modified Look-Locker inversion recovery (MOLLI) sequence for native T1 mapping, and a T2 mapping sequence using T2 prep with bSSFP for myocardial edema. Cine images were acquired in three long axes (2-, 3-, and 4-chamber) and consecutive short-axis views covering the LV from the base to the apex (TR = 3.4 ms, TE = 1.3 ms, flip angle 50°, FOV 320 × 340 mm^2^, matrix size 256 × 144, slice thickness 8 mm with no gap). LGE images were acquired 15 min after intravenous administration of 0.15 mmol/kg gadopentetate dimeglumine (Bayer Healthcare, Berlin, Germany) by using the inversion recovery technique in the identical views (TR = 700 ms, TE = 1.56 ms, flip angle 20°, matrix 256 × 144). The inversion time (TI) was individually optimized to null the normal myocardial signal by using a TI scout sequence.

#### T1 mapping

T1 mapping was acquired in three short-axis slices (basal, mid, and apical) by using a MOLLI sequence (Siemens Healthineers, works-in-progress method 448) with a fixed 5(3)3/4(1)3(1)2 sampling scheme for pre- and post-contrast mapping, respectively. The parameters were as follows: non-selective inversion pulse, bSSFP single-shot readout with 35° excitation flip angle, minimum inversion recovery time of 100 ms, inversion recovery time increment of 80 ms, TR/TE of 2.5/1.12 ms, FOV of 360 × 272 mm^2^, matrix size of 256 × 144, and slice thickness of 8 mm. Post-contrast T1 mapping was performed 20 min after the injection of gadolinium. The T1 map was generated inline right after each scan.

#### T2 mapping

Data were acquired from LV basal, mid, and apical short-axis slices using the T_2_-prepared single-shot bSSFP technique before the administration of gadolinium. Three single-shot bSSFP images with different T_2_ preparation times (TE_T2P_ = 0 ms, 25 ms, 55 ms) were obtained at end-diastole during one breath-hold. Imaging parameters were as follows: TR = 2.4 ms, TE = 1.0 ms, FA = 70°, FOV = 320–340 × 262–278 mm^2^, matrix of 176 × 144, slice thickness of 8 mm, BW = 1093 Hz/px, and GRAPPA acceleration factor of 2. An optional fast variational non-rigid image registration algorithm was used to compensate for the in-plane motion between images. Finally, a pixel-wise myocardial T2 map was generated inline by using curve-fitting with a two-parameter equation.

### Imaging analysis

Two cardiovascular imaging physicians blinded to clinical data independently analyzed all CMR images. LV and right ventricular (RV) volumes and LVEF and RV ejection fraction (RVEF) were assessed using dedicated commercial CMR software (Medis Suite, Version 3.1, Medis, Leiden, Netherlands). Contouring of ventricles was performed following a standardized protocol according to the Society for Cardiovascular Magnetic Resonance (SCMR) post-processing guidelines [23]. Papillary muscles and trabeculations were excluded from the myocardial mass and included in the ventricular volume. Images were evaluated for myocardial LGE by two experienced CMR readers (LYD with 3 years’ clinical experience, CYC with 8 years’ clinical experience). T1 and T2 mapping images were analyzed using dedicated software (Medis Suite, Version 3.1). Myocardial T1 mapping was measured on the mid-ventricular LV short-axis slice. A few contour corrections were required to exclude epicardial fat and trabeculations or blood pool on the images. The T1 value of the blood pool was determined by manually drawing a region of interest in the LV cavity. The hematocrit (HCT) was obtained on the same day as CMR in normal controls and within 3 days prior to the scanning in patients. It was used to calculate the ECV according to the following equation: ECV = (1 - HCT) × (∆R1_myocardium_ / ∆R1_blood_) [24]. T2 mapping was measured on the same mid-ventricular LV slice as T1 mapping. The LV myocardium was manually contoured by tracing the endocardial and epicardial borders. Trabeculations and epicardial fat were carefully excluded, and only the myocardium was included for analysis.

### Statistical analysis

Kolmogorov-Smirnov tests were used to check continuous variables for normal distribution. Normally distributed variables were described as mean ± SD values and tested with Student’s t tests. Non-normally distributed variables were described as median and quartile values and tested with Mann-Whitney U tests. Categorical variables were described as frequencies and percentiles and tested with Pearson’s Chi-square test or Fisher’s exact tests. A Pearson correlation coefficient was used to analyze the correlations between continuous variables. The area under the curve (AUC) in the receiver operating characteristic (ROC) analysis for LVEF recovery was estimated to determine the optimal cutoff points for ECV and native T1 and T2 to predict LVEF recovery. The cutoff value was defined as the value with the maximum Youden index. Univariate and multivariate Logistic regression analyses were performed to identify indicators associated with LV recovery in PPCM patients. Variables with a *P* value < 0.10 (except for diastolic blood pressure, to avoid the collinearity with systolic blood pressure) in univariate analyses were entered into the multivariate logistic regression model. They were analyzed by the forward stepwise method with entry and removal of *p*-values of 0.05 and 0.10, respectively. The results obtained using the logistic regression models were presented as odds ratios (ORs) and 95% confidence intervals (95% CIs). A major adverse cardiac event (MACE) was defined as readmission for HF or cardiac death. Survival curves for MACEs were plotted with the Kaplan-Meier analysis according to the ECV and compared with the results of the log-rank test. In the subgroup analysis of 10 patients who underwent repeated CMR imaging, comparison of tissue characteristics at follow-up and baseline CMR findings was performed using the paired T-test. A *P* value < 0.05 was considered to indicate statistical significance. All statistical analyses were performed using standard statistical software (SPSS Statistics, Version 24.0, Statistical Package for the Social Sciences, International Business Machines, Inc., Armonk, New York, USA).

## Results

### Clinical characteristics

During the study period, 21 PPCM patients (28.4 ± 5.9 years) were included in our CMR registry database. Among these, 15 patients were primiparas and one had twin fetuses. All had obvious symptoms of HF accompanied by an increased serum N-terminal pro-B-type natriuretic peptide (NT-proBNP) level. Their symptom onset times ranged from the last month up to 5 months postpartum. None had a history of hypertension, preeclampsia, or eclampsia before or during the pregnancy. Seventeen patients underwent a cesarean delivery in this gestation, while the others gave natural birth. All patients presented with a sinus rhythm and only one patient showed a left bundle branch block on ECG. Echocardiography showed an LVEF less than 45% in all patients at the time of diagnosis and a mural thrombus in two patients. In comparison with 20 age-matched normal female patients, the PPCM patients had lower systolic blood pressure (103 ± 9 vs 121 ± 6 mmHg, *P* < 0.001) and a higher heart rate (86 ± 17 vs 77 ± 10 bpm, *P* = 0.042) (Table 1). All PPCM patients were given guideline-directed medical therapy, which included a loop diuretic, angiotensin-converting enzyme inhibitor (ACEI)/angiotensin II receptor blockers (ARB), β-blocker, and antisterone (Table 2).

**Table 1:** Characteristics and CMR findings of PPCM patients and healthy volunteers

	PPCM (*n* = 21)	Normal (*n* = 20)	*P* values
Age, years	28.4 ± 5.9	31.3 ± 8.3	0.207
Female, n (%)	21 (100)	20 (100)	NA
BMI (kg/m^2^)	22.1 ± 4.0	21.3 ± 3.2	0.522
SBP (mmHg)	103.1 ± 9.2	121.1 ± 6.3	< 0.001
DBP (mmHg)	68.1 ± 10.5	71.5 ± 8.5	0.269
HR (beat/min)	86.3 ± 16.5	77.3 ± 10.1	0.042
NYHA class			NA
II/III/ IV, n	3/11/7	–	
Lab			
HCT (%)	40.4 ± 4.3	41.8 ± 3.1	0.262
NT-proBNP (pg/ml)	2189.0 [904.0,3435.0]	–	NA
TnT (ng/L)	10.3 [4.7,16.1]	–	NA
CMR findings			
LVEDVI (ml/m^2^)	154.7 ± 31.6	74.5 ± 12.4	< 0.001
LVEF (%)	26.8 ± 10.6	61.2 ± 3.3	< 0.001
LVMI (g/m^2^)	73.6 ± 16.4	40.8 ± 6.1	< 0.001
RVEDVI (ml/m^2^)	120.7 ± 41.0	70.1 ± 18.1	< 0.001
RVEF (%)	33.9 ± 14.6	56.4 ± 5.9	< 0.001
LGE, n (%)	8 (38.1%)	0	NA
ECV (%)	33.9 ± 5.2	27.1 ± 3.1	< 0.001
Native T1 (ms)	1345 ± 79	1212 ± 32	< 0.001
Post T1 (ms)	451 ± 69	483 ± 49	0.090
T2 (ms)	42.3 ± 3.7	36.8 ± 2.3	< 0.001

Female, NYHA class and LGE were described as frequency. NT-proBNP and TnT were described with medians and quartiles, while other data were presented as mean and standard deviations

*Abbreviations*: *CMR* cardiovascular magnetic resonance, *PPCM* peripartum cardiomyopathy, *BMI* body mass index, *SBP* systolic blood pressure, *DBP* diastolic blood pressure, *HR* heart rate, *NYHA* New York Heart Association, *HCT* hematocrit, *NT-proBNP* N-terminal pro-B-type natriuretic peptide, *TnT* troponin T, *LVEDVI* left ventricular end diastolic volume index, *LVEF* left ventricular ejection fraction, *LVMI* left ventricular mass index, *RVEDVI* right ventricular end diastolic volume index, *RVEF* right ventricular ejection fraction, *LGE* late gadolinium enhancement, *ECV* extracellular volume, *NA* not applicable

**Table 2:** Comparison of CMR findings between LVEF-recovered and -unrecovered PPCM patients

	Recovered (*n* = 10)	Unrecovered (*n* = 11)	*P* values
Age, years	27.5 ± 5.9	29.3 ± 6.0	0.505
BMI (kg/m^2^)	22.2 ± 2.3	21.9 ± 5.3	0.852
SBP (mmHg)	106.8 ± 9.0	99.7 ± 8.5	0.079
DBP (mmHg)	73.4 ± 12.1	63.3 ± 6.0	0.023
HR (beat/min)	89.2 ± 18.7	83.7 ± 14.6	0.461
NYHA class			0.771
II/III/ IV, n	2/5/3	1/6/4	
Lab			
HCT (%)	41.6 ± 3.8	39.5 ± 4.6	0.280
NT-proBNP (pg/ml)	1451.5 [470.0, 3398.5]	2243.0 [1817.0, 5976.0]	0.387
TnT(ng/L)	10.1 [2.8, 14.7]	12.5 [7.2, 25.6]	0.236
CMR findings			
LVEDVI (ml/m^2^)	150.9 ± 24.0	158.1 ± 38.0	0.615
LVEF (%)	29.3 ± 11.2	24.5 ± 10.1	0.317
LVMI (g)	70.4 ± 9.3	76.5 ± 21.0	0.405
RVEDVI (ml/m^2^)	118.8 ± 38.8	122.5 ± 44.6	0.845
RVEF (%)	34.3 ± 15.7	33.5 ± 14.4	0.900
LGE, n (%)	2 (20)	6 (54.5)	0.119
ECV (%)	30.7 ± 2.1	36.8 ± 5.6	0.005
Native T1 (ms)	1301 ± 71	1385 ± 66	0.011
Post T1 (ms)	487 ± 77	418 ± 40	0.018
T2 (ms)	40.6 ± 3.0	43.9 ± 3.7	0.040
Medications			
ACEI/ARB, n (%)	9 (90)	11 (100)	0.476
β blocker, n (%)	10 (100)	9 (81.8)	0.476
Antisterone, n (%)	10 (100)	11 (100)	NA
Diuretic, n (%)	8 (80)	10 (90.9)	0.586

*Abbreviations*: *ACEI* angiotensin-converting enzyme inhibitor, *ARB* angiotensin II receptor blockers, others are the same as in Table 1

### CMR imaging characteristics

PPCM patients demonstrated a larger LV end diastolic volume index (LVEDVI, 155 ± 32 vs. 75 ± 12 ml/m^2^ and RV end-diastolic volume index (RVEDVI, 121 ± 41 vs. 70 ± 18 ml/m^2^, both *p* < 0.001), higher LV mass index (LVMI, 73.6 ± 16.4 vs. 40.8 ± 6.1 g/m^2^, *p* < 0.001), lower LVEF (27 ± 11% vs. 61 ± 3%, *p* < 0.001), and lower RVEF (34 ± 15% vs. 56 ± 6%, *p* < 0.001) in comparison with normal controls. LGE was identified in eight (8/21, 38%) PPCM patients, which included five patients with middle-line patterns, two with diffused patterns, and one with a patchy pattern. Among the 10 LVEF-recovered patients, two were LGE-positive with middle-line patterns, while the unrecovered patients included three with middle-line patterns, two with diffused patterns, and one with a patchy LGE pattern. In T1 and T2 mapping, the patient group showed higher values for ECV (33.9 ± 5.2% vs. 27.1 ± 3.1%, *p* < 0.001), native T1 (1345 ± 79 vs. 1212 ± 32 ms, *p* < 0.001) and T2 (42.3 ± 3.7 vs. 36.8 ± 2.3 ms, *P* < 0.001) than the control group (Table 1). When data from the PPCM patients and normal groups were pooled, LVEF was negatively correlated with ECV (Pearson’s *r* = − 0.714, *p* < 0.001), native T1 (Pearson’s *r* = − 0.833, *p* < 0.001), and T2 (Pearson’s *r* = − 0.649, *p* < 0.001).

### CMR imaging characteristics and associations with patient outcome

**Fig. 1:**
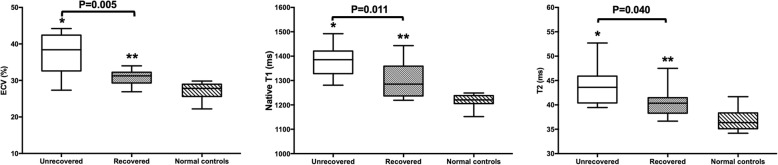
Comparison of native T1/T2 mapping results for the peripartum cardiomyopathy (PPCM) patients and control groups. ECV, extracellular volume. The center line in each box represents the median, whereas the whiskers show the minimum and maximum values. *P < 0.05, unrecovered group vs. normal controls; **P < 0.05, recovered group vs. normal controls

Ten (48%) of the 21 PPCM patients showed LVEF recovery, defined by echocardiography as LVEF ≥50% over at least 6 months’ follow-up. In comparison with the LVEF-unrecovered group, the LVEF-recovered group showed a significantly lower baseline ECV (30.7 ± 2.1% vs. 36.8 ± 5.6%, *P* = 0.005), native T1 (1301 ± 71 vs. 1385 ± 66 ms, *P* = 0.011), and T2 (40.6 ± 3.0 vs. 43.9 ± 3.7 ms, *P* = 0.040) (Fig. 1). However, the LVEDVI, LVEF, LV mass index, RVEDVI, and RVEF at baseline did not show a significant difference between these two groups (all *P* > 0.3). Furthermore, the unrecovered group showed a higher percentage of LGE than the recovered group, although the difference was not statistically significant (54.5% vs. 20%, *P* = 0.12) (Table 2).

We performed ROC analysis and calculated the sensitivity and specificity of native T1, ECV, and T2 in differentiating the LVEF-recovered and LVEF-unrecovered groups. The AUC of ECV for predicting LVEF recovery was 0.83, with an optimal cutoff value of 32.5% (sensitivity, 81.8%; specificity, 90.0%). The AUC of native T1 for predicting LVEF recovery was 0.80, with an optimal cutoff value of 1325 ms (sensitivity, 81.8%; specificity, 70.0%). The AUC of T2 for predicting LVEF recovery was 0.76, and the optimal cutoff value was 43.4 ms (sensitivity, 63.6%; specificity, 90.0%) (Table 3).

**Table 3:** ROC analysis for quantitative tissue characteristics to differentiate between LVEF recovered and unrecovered PPCM patients

Parameters	AUC	Cut-off value	Sensitivity	Specificity
ECV (%)	0.827	32.5	0.818	0.900
Native T1 (ms)	0.800	1325.1	0.818	0.700
T2 (ms)	0.759	43.4	0.636	0.900

*Abbreviations*: *ROC* receiver operating characteristic, *AUC* area under the curve; others are the same as in Table 1

**Fig. 2:**
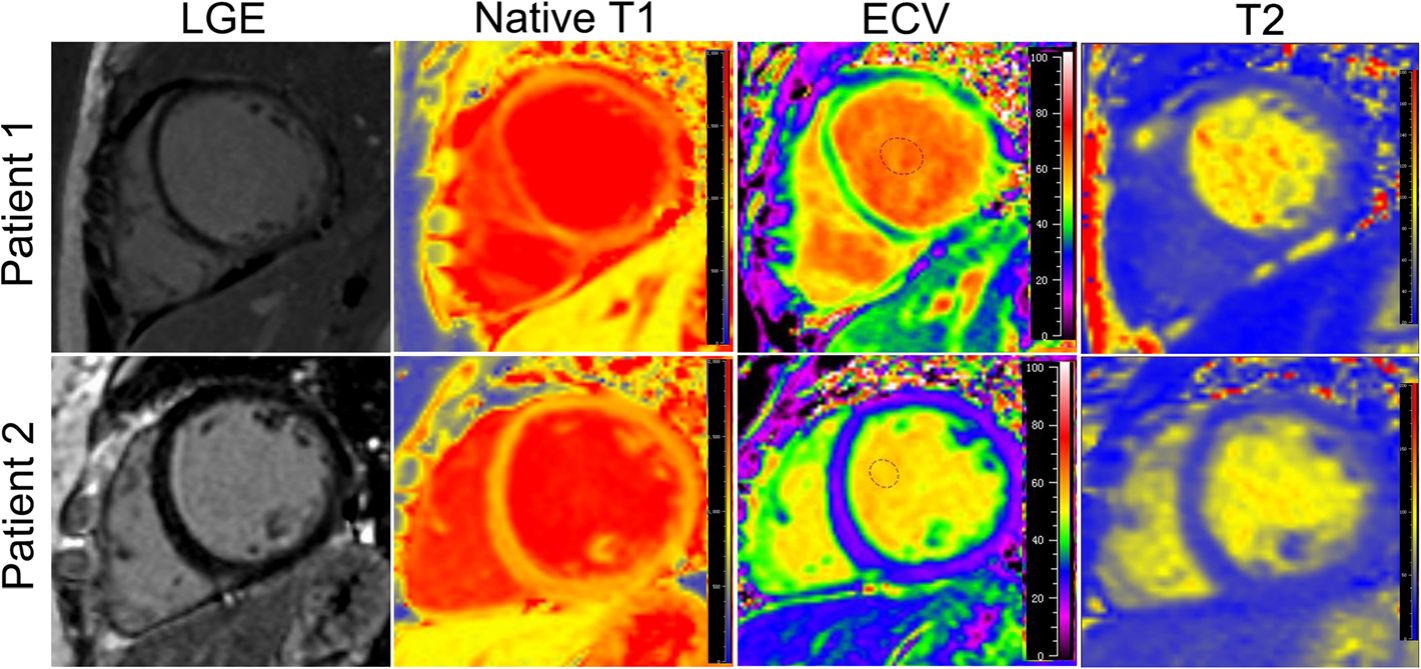
Cardiovascular magnetic resonance (CMR) images for peripartum cardiomyopathy patients. Patient 1, negative late gadolinium enhancement (LGE), native T1 of 1492 ms, extracellular volume (ECV) of 42.8%, T2 of 39.5 ms, unrecovered left ventricular ejection fraction (LVEF); Patient 2, negative LGE, native T1 of 1238 ms, ECV of 26.9%, T2 of 36.7 ms, recovered LVEF

In univariate logistic regression analysis, diastolic blood pressure (OR = 1.15, *P* = 0.045), ECV (OR = 0.70, *P* = 0.029), and native T1 (OR = 0.98, *P* = 0.030) were significantly associated with LV recovery. Systolic blood pressure (OR = 1.11, *P* = 0.097), post-T1 (OR = 1.02, *P* = 0.051), and T2 (OR = 0.71, *P* = 0.063) tended to be associated with LV recovery. In multivariate logistic stepwise analysis, only ECV (OR = 0.58, 95% confidence interval (CI): 0.35–0.96, *P* = 0.032) showed an independent association with LV recovery in PPCM patients (Table 4). Figure 2 shows the LGE, native T1, ECV, and T2 images of two patients who did not show LGE, although one patient showed recovered LV function and the other did not show any recovery in LV function.

**Table 4:** Variables associated with the likelihood of left ventricular recovery in PPCM patients by logistic regression

Variables	Univariable analysis				Multivariable analysis^a^			
	OR	95% CI		*P* values	OR	95% CI		*P* values
		Lower	Upper			Lower	Upper	
Age (years)	0.947	0.813	1.103	0.483				
BMI (kg/m^2^)	1.022	0.822	1.271	0.843				
SBP (mmHg)	1.108	0.982	1.250	0.097	1.216	0.989	1.496	0.064
DBP (mmHg)	1.145	1.003	1.307	0.045				
HR (beat/min)	1.022	0.966	1.082	0.446				
NYHA class	0.672	0.181	2.502	0.554				
logNT-proBNP (pg/ml)	0.408	0.069	2.429	0.325				
logTnT (ng/L)	0.129	0.008	2.108	0.151				
LVEDVI (ml/m^2^)	0.992	0.964	1.021	0.596				
LVEF (%)	1.047	0.960	1.141	0.301				
LVMIi (g)	0.975	0.919	1.033	0.392				
RVEDVI (ml/m^2^)	0.998	0.976	1.019	0.835				
RVEF (%)	1.004	0.946	1.066	0.893				
LGE, n (%)	0.208	0.030	1.467	0.115				
ECV (%)	0.703	0.512	0.965	0.029	0.579	0.351	0.955	0.032
Native T1 (ms)	0.982	0.966	0.998	0.030				
Post T1 (ms)	1.024	1.000	1.048	0.051				
T2 (ms)	0.708	0.492	1.019	0.063				

*CI* confidence interval, *OR* odds ratio. Other abbreviations are the same as in Table 1

^a^All covariates with a *P* value of less than .10 (except for DBP, to avoid the collinearity with SBP) in the univariable analysis were entered into the multivariable model by forward stepwise method

**Fig. 3:**
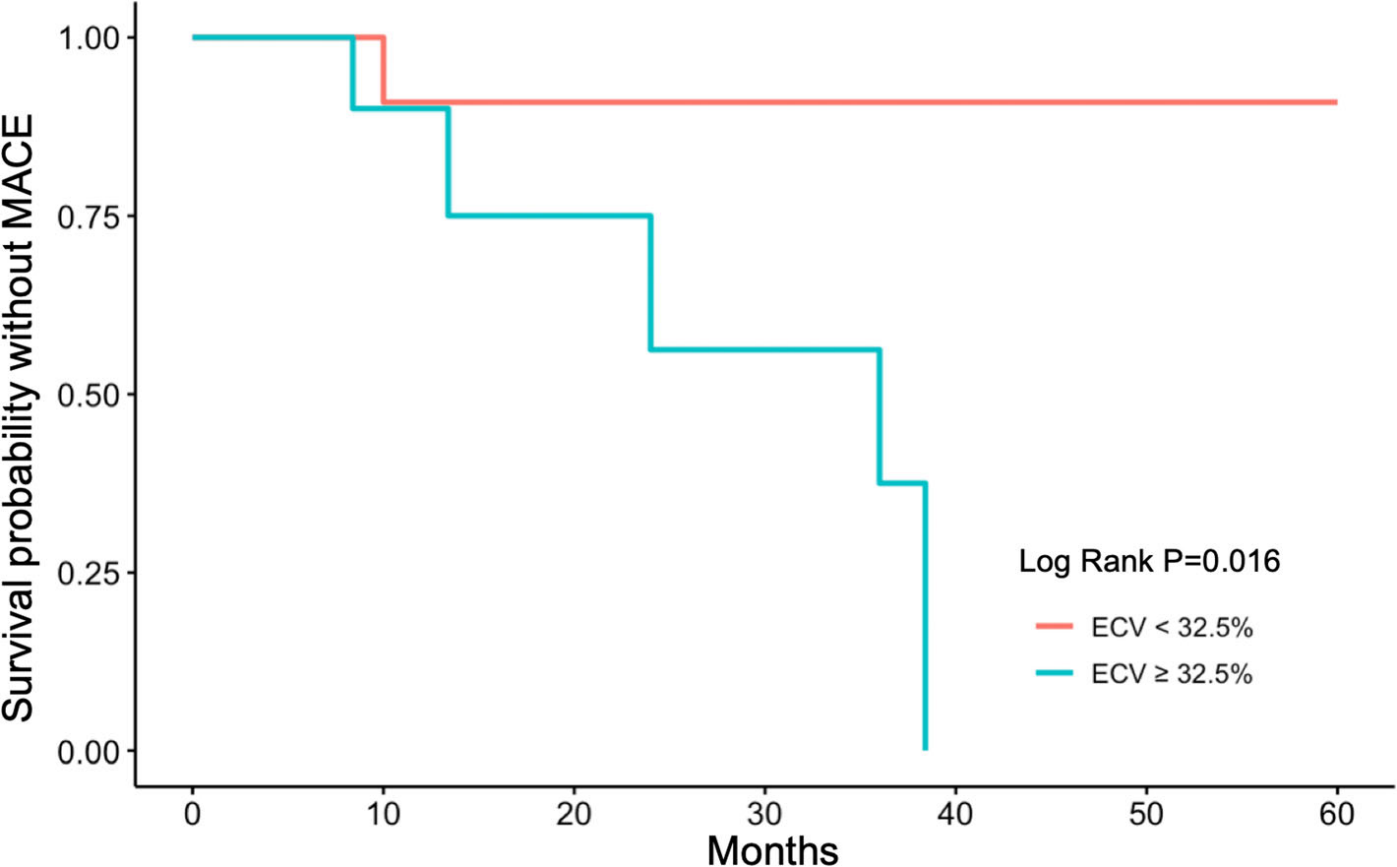
Kaplan-Meier analysis for extracellular volume in clinical outcomes of patients with peripartum cardiomyopathy. ECV, extracellular volume; MACE, major adverse cardiac event, defined as readmission due to heart failure or cardiac death

During the follow-up period (range, 8 months to 5 years; median, 2.5 years), six patients underwent readmission for HF. Among these, one patient showed LVEF recovery after the second readmission and five patients showed no recovery in LVEF. In addition, there were two deaths in the unrecovered group. We performed a Kaplan-Meier survival analysis for MACEs by using the ECV cut-off value of 32.5%. We found that a higher ECV indicated a poor clinical outcome in patients with PPCM (log rank *P* = 0.016, Fig. 3).

### Subgroup analysis of the tissue characteristics in patients with follow-up CMR imaging

Ten patients (27.3 ± 4.9 years) underwent follow-up CMR imaging (median imaging interval 15.3 months, interquartile range: 12.9 to 22.8 months). At baseline, the results of the subgroup were similar to those of all the subjects. Follow-up CMR had a significantly lower native T1 (1367 ± 72 vs. 1289 ± 74 ms, *P* = 0.019 by paired T-test). However, there were no significant differences in ECV and T2 between the CMR imaging performed at baseline and at follow-up. Due to the limited sample size, i.e., five LVEF-recovered patients and five other patients, no significant difference was noted in any variable by T1 and T2 mapping at baseline and follow-up CMR imaging (Table 5).

**Table 5:** The tissue characteristics of PPCM patients by T1 and T2 mapping at baseline and follow-up CMR

	All PPCM (*n* = 10)	Recovered (*n* = 5)	Unrecovered (*n* = 5)	*P* values
Baseline CMR				
ECV (%)	31.9 ± 4.9	29.8 [27.2, 32.6]	35.0 [28.1, 39.1]	0.421
Native T1 (ms)	1367 ± 72	1359 [13106, 1409]	1420 [1281, 1444]	0.690
Post T1 (ms)	451 ± 70	459 [400, 557]	447 [370, 475]	0.421
T2 (ms)	41.8 ± 3.0	39.8 [38.4, 43.8]	42.5 [40.6, 45.3]	0.310
Follow-up CMR				
ECV (%)	30.0 ± 5.8	29.4 [24.1, 34.1]	28.4 [26.0, 36.8]	0.841
Native T1 (ms)	1289 ± 74*	1240 [1205, 1293]	1328 [1273, 1388]	0.056
Post T1 (ms)	432± 71	438 [395, 507]	449 [3400, 476]	0.841
T2 (ms)	40.9 ± 2.8	40.1 [37.1, 44.1]	42.1 [40.0, 42.6]	0.548
Changes between baseline and follow-up CMR				
∆ECV (%)	−2.1 [−7.1, 4.3]	0.3 [−7.6, 5.8]	−2.4 [−7.6, 1.2]	0.548
∆Native T1 (ms)	−65 [−125, −12]	− 110 [− 2010, − 24]	− 23 [−111, 12]	0.222
∆Post T1 (ms)	1.2 [−81.6, 26.6]	1.0 [−80.8, 15.3]	2.7 [−83.9, 53.9]	0.690
∆T2 (ms)	−0.6 [−3.5, 0.9]	−0.5 [−3.2, 2.5]	−2.4 [−3.6, 1.4]	0.690

*P < 0.05, follow-up CMR vs baseline CMR by paired T-test. Delta values are the differences between those parameters’ follow-up value minus baseline value. Abbreviations are the same as in Table 1

## Intra- and inter-observer reproducibility

Both intra-observer and inter-observer reproducibility for assessments of ECV (intra: intraclass correlation [ICC], 0.96 [0.90 to 0.98]; coefficient of variation [COV], 3.01%; inter: ICC, 0.96 [0.90 to 0.98]; COV, 3.20%), native T1 (intra: ICC, 0.96 [0.90 to 0.98]; COV, 1.15%; inter: ICC, 0.94 (0.86 to 0.98); COV, 1.87%), and T2 (intra: ICC, 0.99 [0.97 to 0.99]; COV, 1.20%; inter: ICC, 0.97 [0.93 to 0.99]; COV, 1.63%) were excellent.

## Discussion

To the best of our knowledge, this is the first study reporting quantitative T1 and T2 mapping in PPCM patients. Our findings suggest that CMR native T1 and T2 mapping may be imaging markers with prognostic potential in PPCM. ECV shows an independent association with LV function recovery in PPCM patients.

In our cohort, none of the 21 PPCM patients had hypertension or preeclampsia. Prior studies have reported that hypertension and preeclampsia are risk factors for PPCM [10, 25]. However, a recent review of PPCM indicated that preeclampsia or pregnancy-induced hypertension could also trigger pulmonary edema in the absence of PPCM [1]. Furthermore, one recent study directly compared PPCM with hypertension-associated heart failure in pregnancy (HHFP): the results showed that HHFP was associated with different clinical characteristics (cardiac hypertrophy, a preserved EF, and a better prognosis), indicating that the presence of hypertension in pregnancy-associated HF may not indicate PPCM [26]. We are uncertain why none of the patients in our study had hypertension, unless it is related to racial differences [10], the small sample size, or other unknown reasons, although all 21 cases met all of the diagnostic criteria for PPCM.

Unlike previous studies in which the initial LVEF was reported to be a good predictor of LVEF recovery [10, 27, 28], we did not find that the LVEF-recovered group had significantly higher initial LVEFs than those in the unrecovered group. Previous studies have shown that PPCM is associated with higher rates of thromboembolism than other forms of cardiomyopathy [29, 30]. In addition, many studies have shown that a ventricular thrombus in a PPCM patient is an indicator of poor recovery of LV function [31, 32]. One of the two patients with LV thrombi in our study had unrecovered LV function. Although another patient eventually showed LV recovery, she was readmitted due to signs and symptoms of HF.

In the few previously published CMR studies of PPCM with limited sample sizes, the presence of LGE was likely to predict poor clinical outcomes [14, 15, 19], while our study showed that eight of the 21 PPCM patients had LGE and the unrecovered group had a higher percentage of LGE. However, a recent multicenter study of 34 PPCM patients did not demonstrate the value of focal non-ischemic LGE, which occurred in 71% of the study population [33]. In contrast, another multicenter cohort study reported a very low prevalence of LGE (only 5%) among 40 PPCM patients [16]. Compared to LGE, the T1 mapping technique may show better performance in quantifying the degree of extracellular matrix or interstitial expansion, particularly in pathologies where the differences between normal and affected myocardium are less obvious [34]. Native T1 can be obtained without the use of gadolinium-based contrast, while ECV can detect early fibrosis changes not always detectable by LGE [35]. Previous studies have shown that native T1 and ECV have good predictive value in dilated cardiomyopathy [34, 36], perhaps better than LGE quantification or mid-wall LGE [37]. Both native T1 and ECV correlated well with the histological collagen volume, which may provide reproducible information on diffuse fibrosis [38]. PPCM and idiopathic dilated cardiomyopathy share some genetic [39] and clinical characteristics, and thus, T1 mapping might be adopted in a similar scenario. We evaluated ECV in PPCM, and the unrecovered group showed a significantly higher value. Moreover, ECV showed an independent association with the LV recovery in PPCM. In addition, Kaplan-Meier analysis revealed that patients with a higher ECV were at a higher risk of MACEs. A recent CMR study with T1 mapping showed that during normal pregnancy, there is no elevated native T1 [21]. In our study, LV native T1 was obviously higher than that in normal controls, which indicated that the myocardial characteristics had changed in PPCM. Regarding the subgroup analysis of the 10 PPCM patients who underwent follow-up CMR imaging, a significant decrease was noted in the dynamic change in native T1 at followup. This may indicate an improvement in the myocardial tissue characteristics of PPCM patients. However, due to the small sample size, only a trend rather than statistical significance was observed between LV-recovered and unrecovered groups among those who underwent serial CMR imaging. Further studies on the dynamic changes in myocardial tissue characteristics with a larger sample size are needed.

PPCM is considered to be a form of cardiomyopathy accompanied by inflammation and oxidative stress [40–42]. One case series of CMR findings in PPCM showed that myocardial inflammation in the acute stage was demonstrated by the T2 ratio [18], whereas another case series showed no elevation of the T2 ratio in the acute stage, with only 1 of 10 follow-up images showing an elevated T2 ratio [14]. We also found T2 evaluations according to T2 mapping were significantly higher, indicating the presence of edema, in the PPCM group than in the control group. Due to the inherent limitations of the T2 weighted-image method, T2 mapping is preferred to identify edema of the myocardium [20]. Meanwhile, we found that the LVEF-unrecovered group had a higher T2 than the recovered group. This indicates that the degree of inflammation may be associated with PPCM prognosis.

Previous studies have suggested that gamma globulin has a therapeutic effect on the improvement of LVEF in PPCM patients [42]; however, the exact efficacy of gamma globulin remains controversial [43]. In the present study, we treated PPCM patients with the guideline-directed medical therapy for HF patients, which includes ACEI/ARB, β-blockers, aldosterone, diuretics, and warfarin. Since immunoglobulins were not included in the current study, we could not evaluate their efficacy. In addition, we believe that an early risk assessment of whether a PPCM patient might relapse or not mainly helps doctors identify patients who need more long-term follow-up and greater clinical attention. Hence, predictors of CMR imaging are of great significance for clinical decision-making and communicating with PPCM patients and their families.

## Limitations

This study has several limitations. First, the small sample size may preclude conclusions or extrapolations to a wider patient population. Second, due to ethical concerns, age-matched healthy females were chosen as the control group, rather than women with a normal pregnancy. Third, not all CMR examinations were performed in the acute phase, which may affect T2 value measurement. A more comprehensive assessment of the role of CMR in PPCM through a large, multicenter prospective study with acute-phase and follow-up CMR examinations is needed.

## Conclusions

Native T1, ECV, and T2 were higher in PPCM patients than in normal controls.Our results suggest that native T1, ECV, and T2 may be imaging markers with prognostic utility in PPCM. ECV is associated independently with LV recovery in PPCM patients.

## Data Availability

Data will be available upon request from the corresponding author.

## Abbreviations

ACEI: Angiotensin converting enzyme inhibitor
ARB: Angiotensin II receptor blocker
AUC: Area under the curve
bSSFP: Balanced steady state free procession
CMR: Cardiovascular magnetic resonance
ECG: Electrocardiogram
ECV: Extracellular volume
HCT: Hematocrit
HF: Heart failure
HHFP: Hypertension-associated heart failure in pregnancy
LGE: Late gadolinium enhancement
LV: Left ventricle/left ventricular
LVEDVI: Left ventricular end diastolic volume index
LVEF: Left ventricular ejection fraction
LVMI: Left ventricular mass index
MACE: Major adverse cardiac event
MOLLI: Modified Look–Locker inversion recovery
NT-proBNP: N-terminal pro-B-type natriuretic peptide
PPCM: Peripartum cardiomyopathy
ROC: Receiver operating characteristic
RV: Right ventricle/right ventricular
RVEDVI: Right ventricular end diastolic volume index
RVEF: Right ventricular ejection fraction
SCMR: Society for Cardiovascular Magnetic Resonance
TI: Inversion time
